# A nomogram based on genotypic and clinicopathologic factors to predict the non-sentinel lymph node metastasis in Chinese women breast cancer patients

**DOI:** 10.3389/fonc.2023.1028830

**Published:** 2023-04-19

**Authors:** Liling Zhu, Ke Liu, Baoshi Bao, Fengyun Li, Wentao Liang, Zhaoyun Jiang, Xiaopeng Hao, Jiandong Wang

**Affiliations:** ^1^ Department of Breast Surgery, Breast Tumor Center, Sun Yat-sen Memorial Hospital, Sun Yat-sen University, Guangzhou, China; ^2^ Academic Department of Breast Cancer Education Association, Beijing, China; ^3^ Department of General Surgery, The First Medical Center of the General Hospital of the People’s Liberation Army of China, Beijing, China; ^4^ Academic Department of Beijing Centragene Technology Co., Ltd., Beijing, China

**Keywords:** nomogram, genotypic factor, clinicopathologic factor, non-sentinel lymph node metastasis, breast cancer

## Abstract

**Background:**

Sentinel lymph node biopsy (SLNB) is the standard treatment for breast cancer patients with clinically negative axilla. However, axillary lymph node dissection (ALND) is still the standard care for sentinel lymph node (SLN) positive patients. Clinical data reveals about 40-75% of patients without non-sentinel lymph node (NSLN) metastasis after ALND. Unnecessary ALND increases the risk of complications and detracts from quality of life. In this study, we expect to develop a nomogram based on genotypic and clinicopathologic factors to predict the risk of NSLN metastasis in SLN-positive Chinese women breast cancer patients.

**Methods:**

This retrospective study collected data from 1,879 women breast cancer patients enrolled from multiple centers. Genotypic features contain 96 single nucleotide polymorphisms (SNPs) associated with breast cancer susceptibility, therapy and prognosis. SNP genotyping was identified by the quantitative PCR detection platform. The genetic features were divided into two clusters by the mutational stability. The normalized polygenic risk score (PRS) was used to evaluate the combined effect of each SNP cluster. Recursive feature elimination (RFE) based on linear discriminant analysis (LDA) was adopted to select the most useful predictive features, and RFE based on support vector machine (SVM) was used to reduce the number of SNPs. Multivariable logistic regression models (i.e., nomogram) were built for predicting NSLN metastasis. The predictive abilities of three types of model (based on only clinicopathologic information, the integrated clinicopathologic and all SNPs information, and integrated clinicopathologic and significant SNPs information) were compared. Internal and external validations were performed and the area under ROC curves (AUCs) as well as a series of evaluation indicators were assessed.

**Results:**

229 patients underwent SLNB followed by ALND and without any neo-adjuvant therapy, 79 among them (34%) had a positive axillary NSLN metastasis. The LDA-RFE identified the characteristics including lymphovascular invasion, number of positive SLNs, number of negative SLNs and two SNP clusters as significant predictors of NSLN metastasis. Furthermore, the SVM-RFE selected 29 significant SNPs in the prediction of NSLN metastasis. In internal validation, the median AUCs of the clinical and all SNPs combining model, the clinical and 29 significant SNPs combining model, and the clinical model were 0.837, 0.795 and 0.708 respectively. Meanwhile, in external validation, the AUCs of the three models were 0.817, 0.815 and 0.745 respectively.

**Conclusion:**

We present a new nomogram by combining genotypic and clinicopathologic factors to achieve higher sensitivity and specificity comparing with traditional clinicopathologic factors to predict NSLN metastasis in Chinese women breast cancer. It is recommended that more validations are required in prospective studies among different patient populations.

## Introduction

Breast cancer is a world wild health problem and counts for the 2nd most common causes of cancer death of female cancer survivors (1). USA estimated new breast cancer cases in 2022 is about 290,560 cases and the 5-year breast cancer relative survival rate is 90.0% (2).Meanwhile, there are 429,105 new cases of breast cancer were diagnosed on 2022 (3). The 5-year survival rate is high up to 98.9% if the tumor confined within the breast without the regional or distant diseases. However, 30% of the new cases diagnosed concordant with the regional disease, spreading to regional lymph nodes, and the 5-year survival rate fall down to 85.7% (4). Staging the axillary lymph node (ALN) precisely become a key point to all the breast surgeons.

The length of the survival time is getting longer and longer due to the powerful multidisciplinary treatments. As known to us, the axillary surgery is not just a staging procedure but also an important prognostic factor for recurrence and survival (1, 2), as well as forms the basis for therapeutic decisions (3). The tumor burden information of axilla can be detected through the axillary lymph node dissection (ALND). However, the ALND increases the risk of complications such as lymphedema to 7-14%, shoulder abduction deficits to 75%, numbness to 49% and tingling to 23%, which reduces the quality of life in patients (4, 5). Hence, patients with clinically negative ALNs are unlikely to receive any additional benefit from ALND.

The axillary surgery as an integral part of the breast cancer locoregional surgery, the recent tendency has shifted from the most extended dissection to the minimal invasive procedures as the time passed by. The concept of sentinel lymph node (SLN) was introduced by Zeidman on 1954, which they described the tumor cells constantly spread to the sentinel lymph nodes (6). Due to the metastatic pattern, the sentinel lymph node biopsy was considered as an anatomically reasonable operation. If the SLN is negative, the ALND would be omitted. The ACSOG-Z0011 trial demonstrated 1-2 SLN positive patients who underwent breast conserving surgery can omit the ALND as well (7). Nevertheless, the previous studies reported about 40-75% (8–12) of the SLNB followed by ALND patients were not suffered from the additional lymph node diseases, which means the non-sentinel lymph nodes (NSLN) negative, indicating that these patients underwent unnecessary ALND.

SLNB as the gold standard of the axillary surgery and the ACSOG-Z0011 trial brought up a new scenario to all the breast surgeons. The NSLN metastasis predictive models would help us to evaluate the status of axilla for patients with SLN positive. Most of the models would employ some post-operative indexes such as tumor grade, lymphovascular invasion (LVI) and size of SLN metastasis which were difficult to obtain before surgery (8, 9, 13). CK19 (14) and Maspin (15) mRNA were included into the breast cancer NSLN metastasis predictive models. As the genetic assay developed, high throughput genetic information of the cutaneous melanoma were obtained to train the NSLN (16) metastasis predictive models. As the breast cancer is a composite solid tumor, the genetic variations should make contribution to the regional additional diseases. We first time to employ 96 single nucleotide polymorphisms (SNPs) to analysis the how does the genetic information predict the NSLN tumor burden and take place of the post-operative clinical factors.

## Materials and methods

### Patient recruitments and experiments

This retrospective study collected data from 1,879 women breast cancer patients enrolled from multiple centers. The patients were recruited by the project of “China breast cancer gene mutation hot spot screening clinical multi center research”, which was initiated by China Medical Education Association has been registered in China Clinical Trial Registration Center (Registration Number: ChiCTR180014423). The project is led by the People’s Liberation Army General Hospital, it is responsible for collecting the plasma, monocyte, whole blood, normal tissue, paracancer tissue, cancer tissue and clinical information of breast cancer patients in each sub center. It has collected samples and clinical electronic medical records (EMRs) from sub centers (hospitals) in Beijing, Inner Mongolia, Heilongjiang, Jilin, Liaoning, Hebei, Hunan, Shanxi, Shandong, Shaanxi, Gansu, Jiangsu, Zhejiang, Sichuan and other provinces and regions that have passed the ethics and signed the cooperation agreement. The inclusion criteria:

female patients aged 18-70 years; first diagnosed as invasive breast cancer; tumor samples (including puncture, minimally invasive, cut biopsy, operation) and blood samples can be obtained; complete pathological report can be obtained; patients sign informed consent. The exclusion criteria:DCIS, LCIS, lobar tumor; failure to obtain tumor samples (including puncture, minimally invasive, cut biopsy, surgery) and/or blood samples; patients did not sign informed consent.

In this work, 28 clinicopathologic characteristics were selected from EMR data by breast surgeon and all were converted to categorical variables: (1) Age (≤45 = 0, >45 = 1), (2) BMI (Body Mass Index) (<18.5 = 0, [18.5, 24) = 1, [24, 28) = 2, ≥28 = 3), (3) History of oral contraceptives (no = 0, yes = 1), (4) Smoking history (never = 0, have quit = 1, still = 2), (5) Drinking history (never = 0, have quit = 1, still = 2), (6) Age of menarche (≤12 = 0, (12, 14] = 1, (14, 16] = 2, (16, 18] = 3, >18 = 4), (7) Menstrual cycle (≤27 = 0, (27, 28] = 1, >28 = 2), (8) Menopause (no = 0, yes = 1), (9) Age of menopause (no = 0, >55 = 1, (50, 55] = 2, (45, 50] = 3, (40, 45] = 4, ≤40 = 5), (10) Childbearing history (no = 0, yes = 1), (11) Previous history of breast cancer (no = 0, yes = 1), (12) Previous history of other breast diseases (no = 0, yes = 1), (13) History of ovarian surgery (no = 0, yes = 1), (14) Previous history of tumor (no = 0, yes = 1), (15) Family history of breast cancer (no = 0, yes = 1), (16) Have any close relatives had cancer other than breast cancer (no = 0, yes = 1), (17) cN stage (N0 = 0, N1 = 1), (18) Pathology size (cm) (≤2 = 0, (2, 5] = 1, >5 = 2), (19) Estrogen receptor status (negative = 0, positive = 1), (20) Progesterone receptor status (negative = 0, positive = 1), (21) HER2 status (negative = 0, positive = 1), (22) Subtype (HR+/HER2+ = 0, HR+/HER2- = 1, HR-/HER2+ = 2, TNBC (Tripple Negative Breast Cancer)= 3), (23) pT stage (T1 = 0, T2 = 1, ≥T3 = 2), (24) Pathology subtype (ductal = 0, lobular = 1, mixed = 2, other = 3), (25) Number of total SLNs (1 = 0, 2 = 1, 3 = 2, >3 = 3), (26) Number of positive SLNs (0 = 0, 1 = 1, 2 = 2, ≥3 = 3), (27) Number of negative SLNs (0 = 0 [1, 2], = 1, ≥3 = 2), (28) Proportion of positive SLNs (0 = 0, (0, 0.2] = 1, (0.2, 0.65] = 2, >0.65 = 3).

Genotypic features contain 96 SNPs associated with breast cancer. These 96 SNP variants were carefully selected based on 40 peer-reviewed published articles from PubMed (see **Supplementary Table S1** for details). 54 SNP variants (56%) associated with breast cancer susceptibility were obtained from genome-wide association studies (GWAS) on the risk of breast cancer, 16 SNP variants (17%) associated with chemotherapy or radiation efficacy and toxicity were based on pharmacogenomics and enzyme activity studies, and 26 SNP variants (27%) associated with breast cancer progression, recurrence or metastasis risk rate were discovered through means like survival analysis.

Genomic DNAs of all of fresh breast cancer tissues were isolated by Tissue DNA Extraction Kits (TIANGEN BIOTECH CO., BEIJING, CHINA, cat. DP341-02) (see **Supplement Appendix** section 1.1 for details). SNPs genotyping information were identified by the quantitative PCR detection platform. Bio-Mark™ the Juno 96.96 Genotyping IFC (Fluidigm, US) was used for SNP genotyping (see **Supplement Appendix** section 1.2 for details). Polymerase chain reaction (PCR) primers of the 96 SNPs are listed in **Supplementary Table S2**.

### Statistical analysis

The 96 SNPs were processed through dimensionality reduction by t-distributed Stochastic Neighbor Embedding (t-SNE) (17), with clustering by Density-Based Spatial Clustering of Applications with Noise (DBSCAN) (18), based on the distribution of the patients’ genotypes across the study cohort. Wilcoxon rank-sum test was used to test the significance of difference on the allele frequencies (AFs) of SNPs in different clusters, and Wilcoxon signed-rank test was used to test the significance of difference on the SNP AFs between breast cancer cohort of this study and healthy cohorts from the 1000 Genomes (19) and gnomAD (20) databases. The KEGG (21) pathway enrichment analysis for each SNP cluster was performed. To investigate the association between the risk of NSLN metastasis and the combined effects of SNPs in each cluster, the polygenic risk score (PRS) (22) of each SNP cluster was calculated for every sample using the formula:


(1) $$\text{PRS}=\sum_{i=1}^{n}\beta_{i}x_{i}=\beta_{1}x_{1}+\beta_{2}x_{2}+\ldots \beta_{i}x_{i}+\ldots +\beta_{n}x_{n},$$

where *βi* is the log odds ratio of any given SNP *i* associated with NSLN metastasis (i.e., the coefficient of any given SNP *i* in univariate logistic regression analysis), *xi* is the code of mutation status for the same SNP (0 = wild type and 1 = variant), and *n* is the total number of SNPs in each cluster.

Then, the PRS was normalized by the sigmoid function:


(2) $$p=\frac{1}{1+\text{exp}\{-\left(PRS\right)\}}.$$

Thus, the risk value *p* summarizes the total susceptibility burden of the SNP cluster.

**Figure 1:**
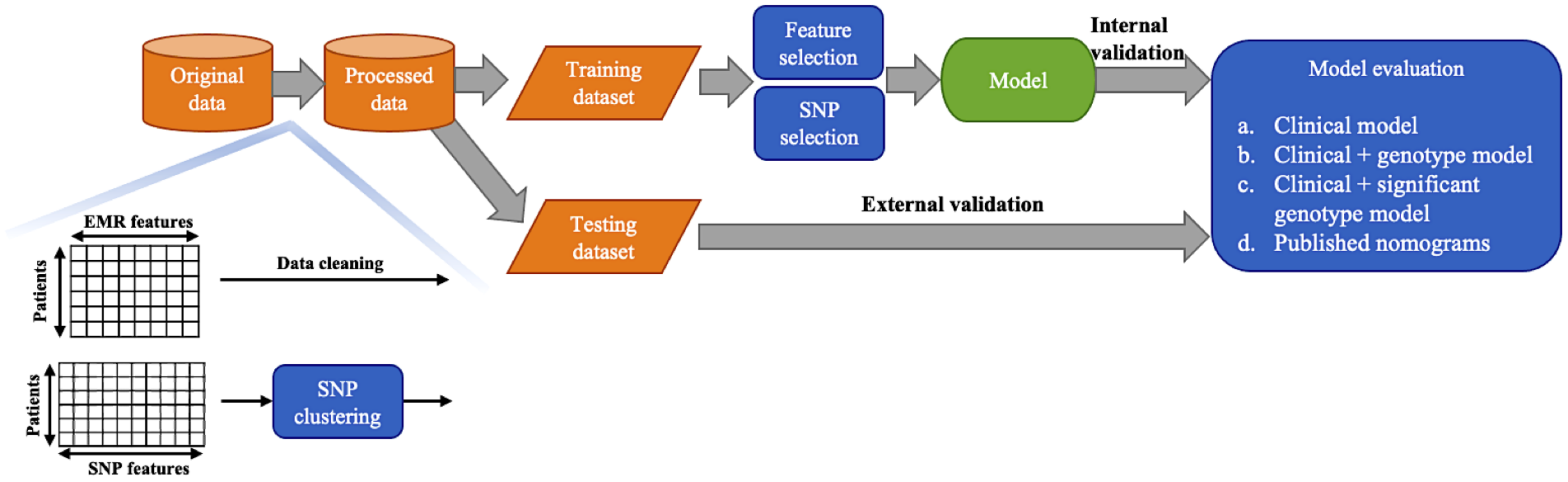
Workflow of this study.

Samples with missing values were removed. In this cohort, 229 patients underwent SLNB followed by ALND and without any neo-adjuvant therapy. 80% (n = 183) patients were randomly sampled as the training dataset and the remaining 20% (n = 46) as the independent testing dataset for external validation. The workflow for selecting features and SNPs, construct and validate the models is summarized in **Figure 1**. Near zero-variance features which identified using the R package ‘caret’ and highly correlated features (spearman correlation coefficient > 0.8) were deleted. The recursive feature elimination (RFE) based on linear discriminant analysis (LDA) was adopted to select the most useful predictive features. In each round of validation, LDA models were trained on training set using candidate features, which were recursively eliminated according to the absolute value of their coefficients on the linear discriminant dimension. Furthermore, the RFE based on support vector machine (SVM) with a sigmoid kernel was used to reduce the number of SNPs. The importance of SNPs for NSLN metastasis were ranked by p-values of Wilcoxon rank-sum test. Multivariable logistic regression models (i.e., nomogram) were built for predicting NSLN metastasis. The predictive abilities of three types of model (based on only clinicopathologic information, the integrated clinicopathologic and all SNPs information, and integrated clinicopathologic and significant SNPs information) were compared. The risk for NSLN metastasis in our dataset was also calculated in the basis of the strategies of ten published models, including MSKCC (23), Cambridge (24), Stanford (25), Mayo (26), MOU (27), Ljubljana (28), MDA (29), Louisville (30), SNUH (31) and Tenon (32) models, but in the absence of features like the size of SLN metastasis, LVI, tumor grade and multifocality. Three random runs of 5-fold cross validation and external validation were performed to evaluate the robustness and the predictive results of the models. By using the receiver operating characteristic (ROC) curve method and various threshold of the predicted probability to distinguish positive and negative NSLN metastasis depending on the point closest to the top-left part of the ROC curve plot with perfect sensitivity or specificity, the accuracy, sensitivity, specificity, false omission rate (FOR), false discovery rate (FDR), F1 score and areas under the ROC curve (AUC) were assessed. All statistical analyses were conducted with R software (version 3.6.2).

Data sharing on Github: https://github.com/gilbertfeng2023/BreastCancerSNP.

## Results

### Clinical characteristics of patients

The analysis cohort consisted of 1,879 patients. In this cohort, 229 patients underwent SLNB followed by ALND and without any neo-adjuvant therapy, 79 (34%) among them had a positive axillary NSLN metastasis. We randomly sampled 80% (n = 183) as the training dataset and the remaining 20% (n = 46) as the independent testing dataset for external validation. We removed six variables that were near zero-variance and two variables that were highly correlated with others. **Table 1** shows the rest 20 descriptive characteristics of the training dataset populations.

**Table 1:** Descriptive characteristics of training dataset populations.

Variable	Category	Non-SLN metastases absent (n = 120) n (%)	Non-SLN metastases present (n = 63) n (%)	Total (n = 183) n (%)
Age	≤45	36 (30)	26 (41)	62 (34)
	>45	84 (70)	37 (59)	121 (66)
BMI (Body Mass Index)	<18.5	7 (6)	1 (2)	8 (4)
	[18.5,24)	60 (50)	28 (44)	88 (48)
	[24,28)	36 (30)	23 (37)	59 (32)
	≥28	17 (14)	11 (17)	28 (15)
History of oral contraceptives	no	112 (93)	59 (94)	171 (93)
	yes	8 (7)	4 (6)	12 (7)
Age of menarche	≤12	4 (3)	3 (5)	7 (4)
	(12,14]	51 (43)	26 (41)	77 (42)
	(14,16]	49 (41)	23 (37)	72 (39)
	(16,18]	13 (11)	7 (11)	20 (11)
	>18	3 (3)	4 (6)	7 (4)
Menstrual cycle	≤27	37 (31)	18 (29)	55 (30)
	(27,28]	56 (47)	33 (52)	89 (49)
	>28	27 (23)	12 (19)	39 (21)
Age of menopause	no	61 (51)	37 (59)	98 (54)
	>55	3 (3)	0 (0)	3 (2)
	(50,55]	17 (14)	12 (19)	29 (16)
	(45,50]	31 (26)	8 (13)	39 (21)
	(40,45]	7 (6)	4 (6)	11 (6)
	≤40	1 (1)	2 (3)	3 (2)
Previous history of other breast diseases	no	109 (91)	54 (91)	163 (86)
	yes	11 (9)	9 (9)	20 (14)
Family history of breast cancer	no	115 (96)	58 (92)	173 (95)
	yes	5 (4)	5 (8)	10 (5)
Have any close relatives had cancer other than breast cancer	no	103 (86)	57 (90)	160 (87)
	yes	17 (14)	6 (10)	23 (13)
cN stage	N0	104 (87)	50 (79)	154 (85)
	N1	16 (13)	13 (21)	28 (15)
Pathology size (cm)	≤2	67 (56)	30 (48)	97 (53)
	(2,5]	52 (43)	30 (48)	82 (45)
	>5	1 (1)	3 (5)	4 (2)
Estrogen receptor (ER) status	negative	16 (13)	5 (8)	21 (11)
	positive	104 (87)	58 (92)	162 (89)
Progesterone receptor (PR) status	negative	22 (18)	7 (11)	29 (16)
	positive	98 (82)	56 (89)	154 (84)
HER2 status	negative	89 (74)	47 (75)	136 (74)
	positive	31 (26)	16 (25)	47 (26)
Subtype	HR+/HER2+	22 (18)	13 (21)	35 (19)
	HR+/HER2-	82 (68)	45 (71)	127 (69)
	HR-/HER2+	9 (8)	3 (5)	12 (7)
	TNBC	7 (6)	2 (3)	9 (5)
Pathology subtype	ductal	86 (72)	46 (73)	132 (72)
	lobular	6 (5)	3 (5)	9 (5)
	mixed	1 (1)	2 (3)	3 (2)
	other	27 (23)	12 (19)	39 (21)
Number of total SLNs	1	7 (6)	5 (8)	12 (7)
	2	16 (13)	8 (13)	24 (13)
	3	36 (30)	15 (24)	51 (28)
	>3	61 (51)	35 (56)	96 (52)
Number of positive SLNs	1	81 (68)	23 (37)	104 (57)
	2	28 (23)	20 (32)	48 (26)
	≥3	11 (9)	20 (32)	31 (17)
Number of negative SLNs	0	9 (8)	14 (22)	23 (13)
	[1,2]	61 (51)	32 (51)	93 (51)
	≥3	50 (42)	17 (27)	67 (37)
Proportion of positive SLNs	(0,0.2]	23 (19)	5 (8)	28 (15)
	(0.2,0.65]	70 (58)	31 (49)	101 (55)
	>0.65	27 (23)	27 (43)	54 (30)

### SNP clustering

**Figure 2:**
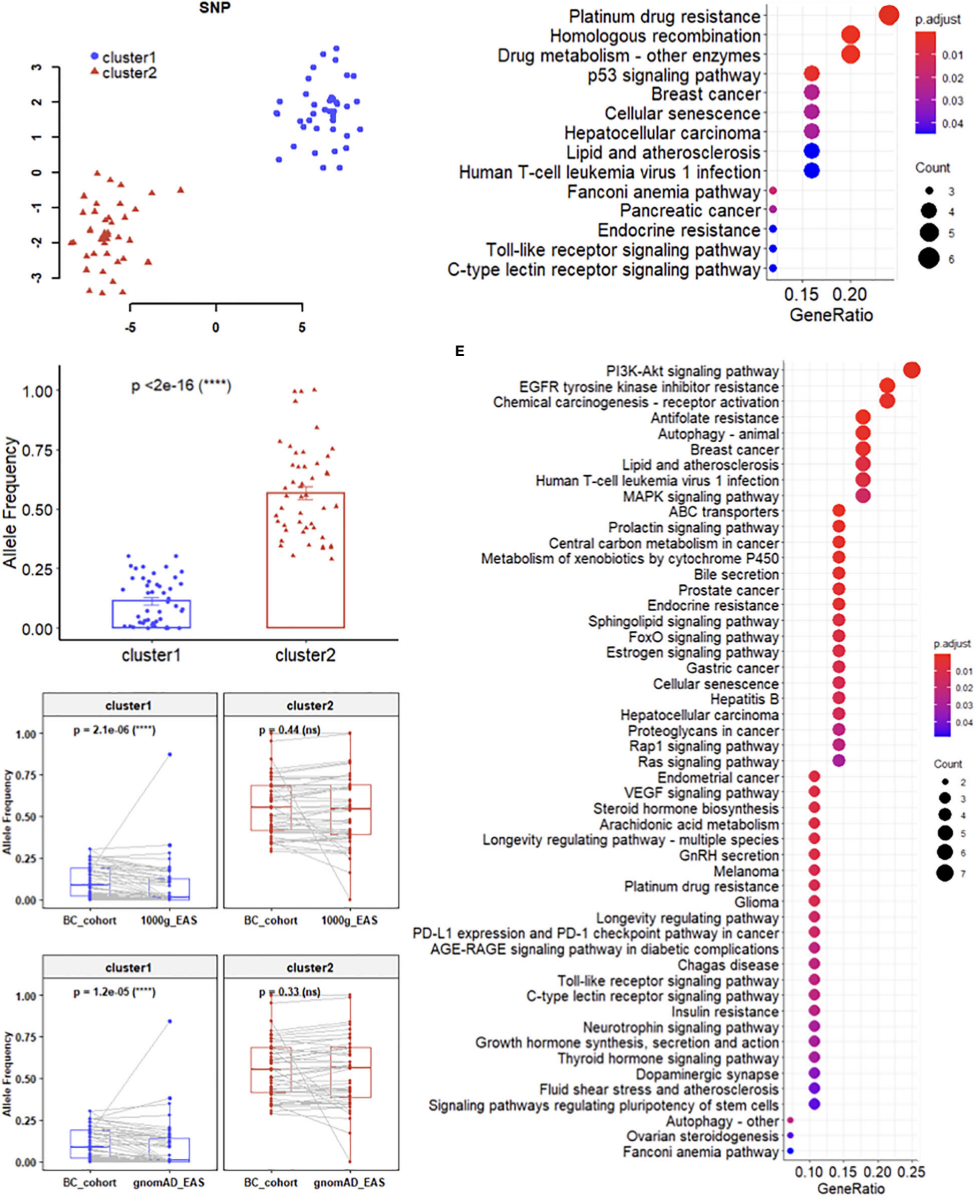
Mutation features of SNP clusFters. **(A)** Dimensionality reduction and clustering of 96 SNPs. **(B)** Difference between the AFs of SNPs from cluster 1 and cluster 2. **(C)** Difference between the AFs of SNPs in breast cancer cohort and healthy cohorts. **(D, E)** KEGG pathway enrichment analysis for cluster 1 **(D)** and cluster 2 **(E)**, respectively.

SNPs should not be considered individually since cancers are generally considered as multigenic diseases. The visualization of dimensionality reduction by t-SNE (17) showed that the 96 SNPs were divided into two clusters in two-dimensional space (**Figure 2A**). Then we used DBSCAN (18) to obtain these two sets of SNPs, which named cluster1 and cluster2 respectively, and each of them contained 48 SNPs. Intuitively, the difference between the two sets of SNPs is their mutational stability in the cohort study. Statistically, the AFs of SNPs from cluster2 were significantly higher than those from cluster1 (**Figure 2B**). Besides, there were more missense mutations distributed in cluster1 (**Figure S1**). In addition, significant difference of the AFs of SNPs in cluster1 were detected between breast cancer cohort of this study and healthy cohorts from the 1000 Genomes (19) and gnomAD (20) databases, but not in cluster2 (**Figure 2C**). The KEGG (21) pathway enrichment analysis revealed that these two clusters involved different pathways (**Figures 2D, E**).

### SNP cluster assignment

To assign the risk value which evaluates the combined effect of each SNP cluster, we firstly calculated the log odds ratio of each SNP associated with NSLN metastasis (i.e., the coefficient of each SNP in univariate logistic regression analysis). 12 SNPs that were around zero-variance in the whole dataset were identified and removed. The coefficients and cluster index of the rest 84 SNPs are listed in **Supplementary Table S3**. Then the risk values of two SNP clusters of every sample were calculated according to equation (1) and (2).

### Feature selection

**Figure 3:**
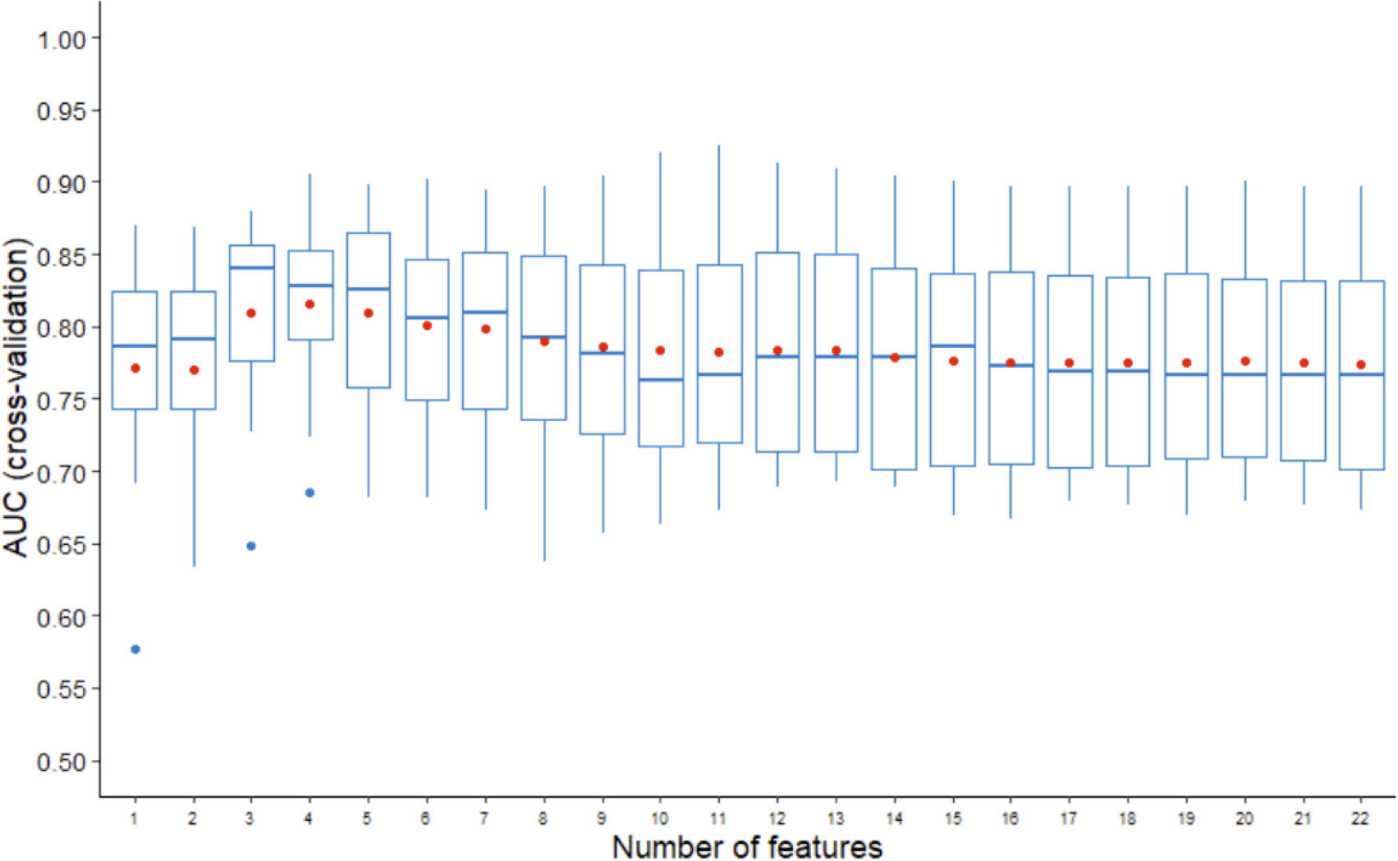
AUCs of three random runs of 5-fold cross validation using LDA-RFE. The line in box represents the median, and the red dot represents the average.

We combined the two SNP cluster features and the clinicopathologic features to construct NSLN metastasis predictive model. The feature selection procedure was completed using the LDA-RFE method. **Figure 3** shows that the top three predictors can achieve the highest median AUC while the top four predictors can achieve the highest average AUC in three random runs of 5-fold cross validation. To avoid an overfitting model, four predictors were chosen. We counted the number of times of the last four features that were recursively eliminated in the 15 rounds of validation, and the top four features from high to low are cluster2 (15 times), cluster1 (15 times), number of positive SLNs (14 times) and number of negative SLNs (7 times). It is worth mentioning that cluster2 was the last feature to be eliminated in each round of validation.

### SNP selection

**Figure 4:**
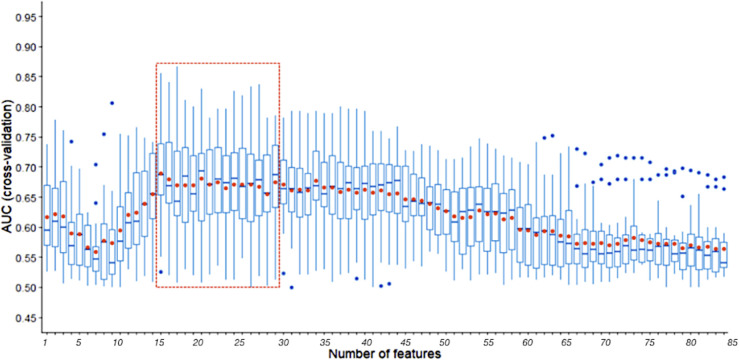
AUCs of three random runs 5-fold cross validation using SVM-RFE with a sigmoid kernel. The line in box represents the median, and the red dot represents the average. The part in the red dotted box is the peak region of median and average AUCs.

Using a small set of SNPs to predict NSLN metastasis reduces the costs associated with assays and will undoubtedly have clinical application. The SVM-RFE identified fewer relevant SNPs which still supplied better predictive performance than all SNPs in three random runs of 5-fold cross validation. As illustrated in **Figure 4**, the median and average AUCs obtained by the top 15-29 SNPs were higher than those obtained by other number of SNPs. The top 29, 20 and 15 SNPs were selected for further model training and validation. The top 29 SNPs are marked in **Table S3**. Among them, 13 were in cluster1 and 16 were in cluster2.

### Model performance validation

**Figure 5:**
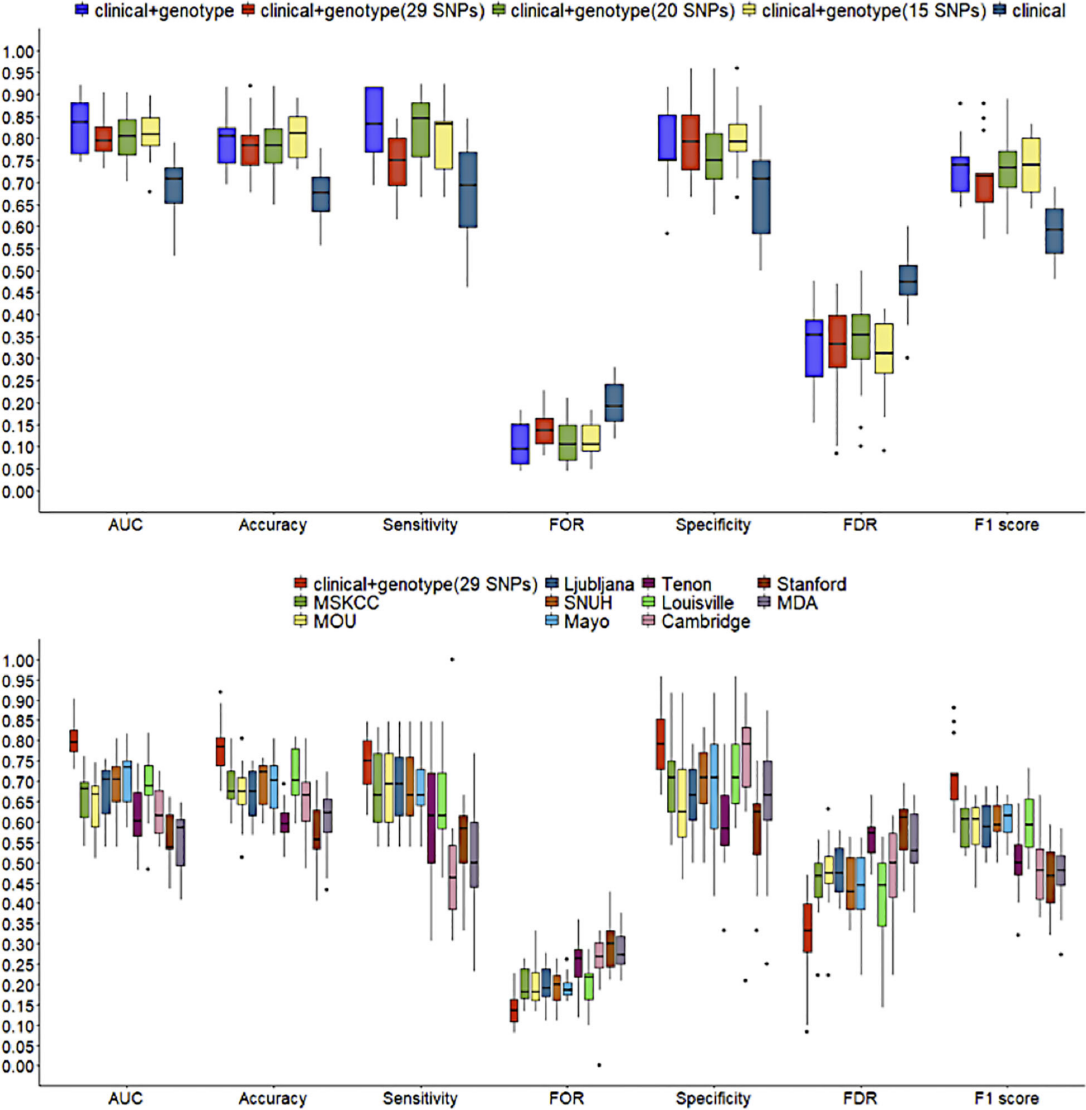
Comparisons of predictive performance in 5-fold cross validation. **(A)** among NSLN metastasis predictive models using different numbers of SNPs, and **(B)** between the clinical and 29 significant SNPs combining model in this study and ten published NSLN metastasis predictive models. The line in box represents the median.

A comparison among the performance of three types of NSLN metastasis predictive model which based on the integrated clinicopathologic and all SNPs information (i.e., the “clinical + genotype” model), the integrated clinicopathologic and significant SNPs information (i.e., the “clinical + genotype (29 SNPs)” model, the “clinical + genotype (20 SNPs)” model, and the “clinical + genotype (15 SNPs)” model) and only clinicopathologic information (i.e., the “clinical” model) respectively was made. In internal validation (**Figure 5A**), the median AUCs of the clinical and all SNPs combining model, the clinical and significant SNPs combining model (including 29, 20 and 15 SNPs), and the clinical model were 0.837, 0.795, 0.804, 0.809 and 0.708 respectively. Meanwhile, in external validation (**Table 2.1**), the AUCs of the five models were 0.817, 0.815, 0.783, 0.785 and 0.745 respectively. This result suggests that the model using 29 SNPs is more robust than the model using 20 or 15 SNPs, which may be overfitting in training dataset.

**Table 2.1:** Predictive performance of different NSLN metastasis predictive models in external validation, among NSLN metastasis predictive models using different numbers of SNPs.

	AUC	Accuracy	Sensitivity	FOR	Specificity	FDR	F1 score
clinical+genotype	0.817	0.826	0.688	0.156	0.900	0.214	0.733
clinical+genotype (29 SNPs)	0.815	0.804	0.750	0.138	0.833	0.294	0.727
clinical+genotype (20 SNPs)	0.783	0.804	0.625	0.182	0.900	0.231	0.690
clinical+genotype (15 SNPs)	0.785	0.739	0.688	0.179	0.767	0.389	0.647
clinical	0.745	0.652	0.938	0.063	0.500	0.500	0.652

Using the training dataset of our study, ten published models, including MSKCC (23), Cambridge (24), Stanford (25), Mayo (26), MOU (27), Ljubljana (28), MDA (29), Louisville (30), SNUH (31) and Tenon (32) models, were reconstructed according to their clinicopathologic variables, but in the absence of features like the size of SLN metastasis, LVI, tumor grade and multifocality. **Figure 5B** and **Table 2.2** shows that the clinic + genotype (29 SNPs) model visibly outperformed than other models, especially on AUC, accuracy and F1 score. It is worth noting that whether in internal validation or external validation, only the combining model was greater than 0.750 on both sensitivity and specificity.

**Table 2.2:** Predictive performance of different NSLN metastasis predictive models in external validation, among different clinical nomogram models.

	AUC	Accuracy	Sensitivity	FOR	Specificity	FDR	F1 score
clinical+genotype (29 SNPs)	0.815	0.804	0.750	0.138	0.833	0.294	0.727
MSKCC	0.736	0.652	0.813	0.150	0.567	0.500	0.619
MOU	0.727	0.630	0.750	0.190	0.567	0.520	0.585
Ljubljana	0.716	0.652	0.813	0.150	0.567	0.500	0.619
SNUH	0.700	0.630	0.875	0.118	0.500	0.517	0.622
Mayo	0.690	0.652	0.875	0.111	0.533	0.500	0.636
Tenon	0.685	0.630	0.750	0.190	0.567	0.520	0.585
Louisville	0.679	0.630	0.688	0.217	0.600	0.522	0.564
Cambridge	0.660	0.652	0.500	0.267	0.733	0.500	0.500
Stanford	0.548	0.543	0.563	0.304	0.533	0.609	0.462
MDA	0.521	0.565	0.438	0.321	0.633	0.611	0.412

### Nomogram of NSLN metastasis

**Figure 6:**
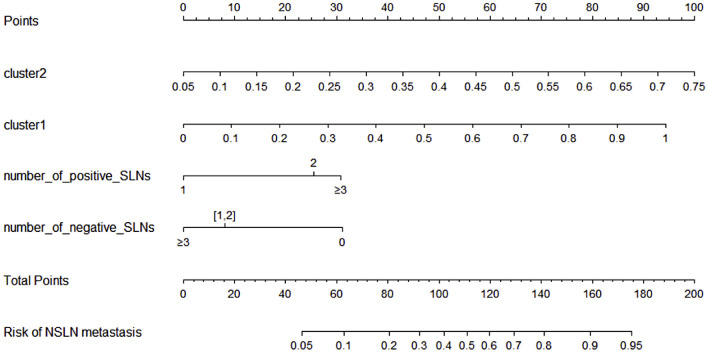
NSLN metastasis predictive nomogram integrated the four predictors selected by LDA-RFE. The first row (Points) is the point assignment for each factor. Rows 2-5 represent the predictors included in the model. For an individual patient, each factor is assigned a point value based on the value range or characteristic. The assigned points for all four factors are summed, and the total is found in row 6 (Total Points). Once the total is located, the predicted risk of NSLN metastasis is confirmed in row 7.

A NSLN metastasis predictive nomogram created based on the “clinic + genotype (29 SNPs)” model and developed in the training population (n = 183) is shown in **Figure 6**. NSLN metastasis predictive nomogram integrated the four predictors selected by LDA-RFE. The first row (Points) is the point assignment for each factor. Rows 2-5 represent the predictors included in the model. For an individual patient, each factor is assigned a point value based on the value range or characteristic. The assigned points for all four factors are summed, and the total is found in row 6 (Total Points). Once the total is located, the predicted risk of NSLN metastasis is confirmed in row 7.

## Discussion

Metastasis of malignant tumors to regional lymph nodes is one of the early signs of cancer spread (33). The presence of cancer cells in regional lymph nodes is a key factor of poor outcome in breast cancer (34). Gene expression analysis in previous studies revealed that gene and pathway profiles underlie lymph node metastasis in breast cancer patients significantly altered (35). NSLN positive and negative patients have distinct features in gene expression variance, which down-regulated genes involved in B cell antigen receptor (BCR) pathway and up-regulated genes associated with ER signaling pathway significantly occurred in the NSLN positive group (35, 36). It has been well demonstrated that the prognostic and predictive factors for breast cancer is a combination of genetic, epigenetic, physiological and environmental factors (37–39).

In this study, we reviewed numerous studies about breast cancer susceptibility and association analysis of prognostic characteristics in breast cancer from peer-reviewed published literatures, including large-scale GWAS or meta-analysis. 96 SNPs were selected from the above studies. We analyzed the genetic testing results of the whole cohort, and discovered these SNP variants mainly distributed in the pathways associated with breast cancer. Such as the p53 is a tumor suppressor gene (40),mutation of p53 was related the worse overall survival of the breast cancer patients. PI3K/AKT/mTOR (41)signaling cascade alteration is highly related to drive the breast cancer cell grow which induced uncontrolled cell growth and proliferation. Another major signaling altered is the MAPK pathway repressed is disturb the balance between the self-renewal, proliferation of the tumor-initiating cell (42). ER signaling pathway is very important of the breast cancer and it’s related to the development, proliferation and the progression of the tumor (43).In order to combine genetic variation features and clinicopathologic characteristics in the prediction of NSLN, we adopted dimensionality reduction and clustering method to generate potential genetic predictors for our models. Based on the allele frequency of the SNPs occurred in the patients, we found the SNPs could be divided to two distinct subgroups/clusters: cluster1 covered the majority of the “rare” variants which mainly associated with tumorigenesis involving the pathway of DNA repair, steroid hormone synthesis and immune deficiency whereas cluster2 covered most of the “common” variants which mainly associated with cancer progression or therapy response involving the pathway of PI3K-AKT signaling, MAPK signaling pathway, estrogen signaling pathway and drug metabolism pathway. Comparing with 1000 Genomes and gnomAD eastern Asian population, which collect healthy women samples, the cluster1 SNPs in our cohort are significantly different by allele frequency, while no statistically significance for the cluster2 SNPs. Such observation implicitly indicates the cluster1 SNPs probably play more important roles in early breast cancer patients in China. In the further mechanism study, these SNPs might need to be paid more attention.

In 15 rounds of LDA-RFE, the two SNP clusters and three clinicopathologic characteristics were influential contributors to the predictive models. The two SNP clusters were the most important predictors across all rounds of feature selection, suggesting the generated SNP clusters based on the distribution of the patients’ genotypes can represent the genotypic contributions on NSLN metastasis prediction in lower dimensions. The selected clinicopathologic characteristics, including the number of positive and negative SLN, have been reported in other predictive models (23, 26–28, 31), denoting these features are important to NSLN metastasis prediction. In fact, these clinicopathologic features are already demonstrated as prognostic factors in breast cancer survival (37). We have integrated the two SNP cluster features and the three clinicopathologic characteristics to establish a more robust NSLN metastasis predictive nomogram. Whether in internal or external validation, the model displayed a better performance than that only use the same clinicopathologic characteristics and those reported in previous studies to predict NSLN metastasis. Therefore, we are optimistic that genotypic factors integrated with clinicopathologic data will facilitate the development of a model superior to the application of traditional clinicopathologic data alone. On the other hand, genetic assay can be promoted as a conventional technical mean used for predicting NSLN metastasis in the future and take place of the post-operative indexes such as nuclear grade (11, 23), histological grade (24), LVI (23, 25, 27–29, 44) and size of SLN metastasis (24–27, 29, 44) which were unlikely to obtain before surgery. For example, it is difficult to assess the LVI status by preoperative core needle biopsy or intraoperative frozen section (45).

Our study further screened the relevant SNPs to NSLN metastasis and reduced the number of SNPs to 29. We had developed a procedure based on SVM-RFE method to accomplish this. Considering the reliability of results and reducing the amount of calculation, we decided to conduct 5-fold cross validation for three random times, i.e., a total of 15 times of recursive SNP elimination, since the number of SNPs selected in two random runs of 5-fold cross validation was 15 (**Figure S2A**), while in four random runs of 5-fold cross-validation it had basically stabilized at 15-29 (**Figure S2B**). Meanwhile, we also tested the effect of SVM-RFE with a radial kernel and a linear kernel, and selected a subset of 10-16 SNPs and 15-19 SNPs respectively (**Figures S2C, D**). According to the external validation results in our work, such size of SNP subsets contains too little information to train a robust model. On the contrary, the subset of 29 SNPs could provide predictive performance comparable to 84 SNPs and was distinctly superior to the clinical model. This was further verified using other six independent testing datasets by randomly sampling (**Supplementary Table S4**). In addition, it will reduce the cost of genetic testing for breast cancer patients, thereby facilitating its daily clinical application in breast cancer management.

The SNPs used in this study were associated with breast cancer, which could be used to predict whether NSLN metastasis occurred for breast cancer patients based on our model combining clinicopathologic factors. However, the biological mechanism of these SNPs in the metastasis of breast cancer remains unclear. To explore the underlying regulatory mechanisms of the 29 SNPs screened in our study in the metastasis of breast cancer, we performed an expression quantitative trait loci (eQTL) analysis (46) using the datasets from eQTLGen (47) database (https://eqtlgen.org/cis-eqtls.html). We conducted cis-eQTL analysis between 29 SNP loci and corresponding gene expression data in eQTLGen datasets. In this database, cis-eQTLs were defined that the gene expression levels were affected by a gene-proximal (<1Mb) SNP (47). 48 cis-eQTL genes for 16 SNPs were found (**Table S5**). The results of further deep literature review showed that the expression levels of cis-eQTL genes for 12 SNPs were related to the prognosis or metastasis of breast cancer (**Supplementary Table S5**). The literatures elucidate that the expression levels of some genes are different between breast cancer tissues and normal tissues, some genes are related to the cancer cell metastasis, and some genes are related to the metastasis-related genes. These literature evidence indicate that these SNPs associated with breast cancer also contributed to the metastasis of breast cancer. The eQTL analysis and corresponding literature evidence gave us clues that these SNPs might play their roles by regulating the expression of some genes to affect the metastasis of breast cancer.

## Conclusion

Herein we present a new nomogram by combining genotypic and clinicopathologic factors to achieve higher sensitivity and specificity comparing with traditional clinicopathologic factors to predict NSLN metastasis in Chinese women breast cancer. Unlike the previous published models for NSLN metastasis, our nomogram is more sensitive to the genotypic features and the clinical or pathological features are more easily to be available. However, our nomogram is built using a relatively small sample size. It is recommended that more external validations are required in prospective studies among different patient populations. Furthermore, the eQTL analysis in this study suggested that some polymorphisms might affect breast cancer’s metastasis *via* regulating downstream gene expression, which would be helpful for the deep biological insight of breast cancer in the future.

## Data availability statement

All relevant data is contained within the article. The original contributions presented in the study are included in the article/**Supplementary Material**, further inquiries can be directed to the corresponding author.

## Ethics statement

The studies involving human participants were reviewed and approved by the ethics committee of Chinese PLA General Hospital. The patients/participants provided their written informed consent to participate in this study.

## Author contributions

(I) Conception and design: LZ and BB; (II) Administrative support: JW and XH; (III) Provision of study materials or patients: All authors; (IV) Collection and assembly of data: All authors; (V) Data analysis and interpretation: All authors; (VI) Manuscript writing: All authors. All authors contributed to the article and approved the submitted version.
