# Supplementary material for: A nomogram based on genotypic and clinicopathologic factors to predict the non-sentinel lymph node metastasis in Chinese women breast cancer patients
Source: Front Oncol. 2023 Apr 19;13:1028830. doi: 10.3389/fonc.2023.1028830 (PMC10154525; doi:10.3389/fonc.2023.1028830)
Supplement: Supplementary file 1 [file DataSheet_1.docx]

**A nomogram based on genotypic and clinicopathological factors to predict the non-sentinel lymph node metastases in Chinese women breast cancer patients - Supplementary Appendix**

1. **Experimental implementation**

1.1 DNA isolation

Take 10-50 mg of breast cancer tissue, put it into a centrifuge tube containing zirconium beads, add 200 μl of tissue digestive fluid GHA and 20 μl of proteinase K, and grind it with an electric homogenizer for about 25 s until the tissue is fully ground. Digestion at 65 °C for 90 min to complete digestion. Add 300 μl of cracking liquid GHB, shake and mix. Place the centrifuge tube at 75 °C, incubate for 15 min, and mix it upside down for 3 times, 3-5 times each time. Place at room temperature for 5 min. Add 350 μl isopropanol, shake and mix for 10 sec. Add 30 μl suspension g of magnetic beads, shake and mix for 1 min, leave for 9 min in total, shake and mix for 1 min every 3 min. Place the centrifuge tube on the magnetic frame for 30 sec. After the magnetic bead is completely absorbed, carefully absorb the liquid. Add 700 μl buffer GDA (please check whether anhydrous ethanol has been added before use), shake and mix for 30 seconds. Place the centrifuge tube on the magnetic frame for 30 sec. After the magnetic bead is completely absorbed, carefully absorb the liquid. Add 700 μl of rinse PWD (please check whether absolute ethanol has been added before use), shake and mix for 30 seconds. Place the centrifuge tube on the magnetic frame for 30 sec. After the magnetic bead is completely absorbed, carefully suck the liquid. Repeat steps 10 and 11 once. Place the centrifuge tube on a magnetic stand and dry at room temperature for 10-15 minutes. Remove the centrifuge tube from the magnetic frame, add 100 μl of eluent buffer TB, shake and mix, place it at 56 °C, incubate for 10 min, and mix it upside down for 3 times, 3-5 times each time. Place the centrifuge tube on the magnetic frame for 2 min, after the magnetic beads are completely absorbed, carefully transfer the DNA solution to a new centrifuge tube and store it at -20 ℃.

1.2 SNP genotyping

Review the pipetting map for exact locations to pipet reagents into the IFC. Pipet reagents from the SNP Type assay plate and the sample plate to the IFC according to the 96-well plate locations shown on the pipetting map. Ensure that the notched corner of the IFC (“A1”) is at the top left, and view the loading map at the bottom of the IFC. Load an entire syringe of Juno 96.96 GT Control Line Fluid in Acc1 and a second syringe in Acc2 (pink squares on the pipetting map). Load an entire syringe of Juno 96.96 GT Control Line Fluid into a reservoir and a second syringe into the second reservoir (long pink rectangles on the right side of the pipetting map). Pipet 15 μl of Juno GT Flux Fluid into each of the six ports (purple circles on the pipetting map). Unseal the SNP Type assay plate and pipet 4.0 μl of each assay mix into an assay inlet (black circles on pipetting map). Unseal the sample plate and pipet 4.0 μl of each sample mix into a sample inlet (green circles on the pipetting map). Pull the sticker front tab down and away from the IFC to gently peel off the loading map. Do not invert the IFC. If necessary, remove any bubbles from an IFC inlet by removing the contents by pipette and then carefully re-pipetting the contents into the inlet. Ensure that the SX interface plate (silver label) is installed on the instrument. Start the run <60 minutes after pipetting the reagents into the IFC. On the Juno scripts screen, tap the SNP Type tab, Juno 96.96, then Run. After the IFC is finished, tap EJECT to eject the IFC, then perform an end-point read of the IFC in ≤60 minutes. Do not leave the IFC in the instrument overnight.
