# Supplementary figures and images for: A nomogram based on genotypic and clinicopathologic factors to predict the non-sentinel lymph node metastasis in Chinese women breast cancer patients
Source: Front Oncol. 2023 Apr 19;13:1028830. doi: 10.3389/fonc.2023.1028830 (PMC10154525; doi:10.3389/fonc.2023.1028830)

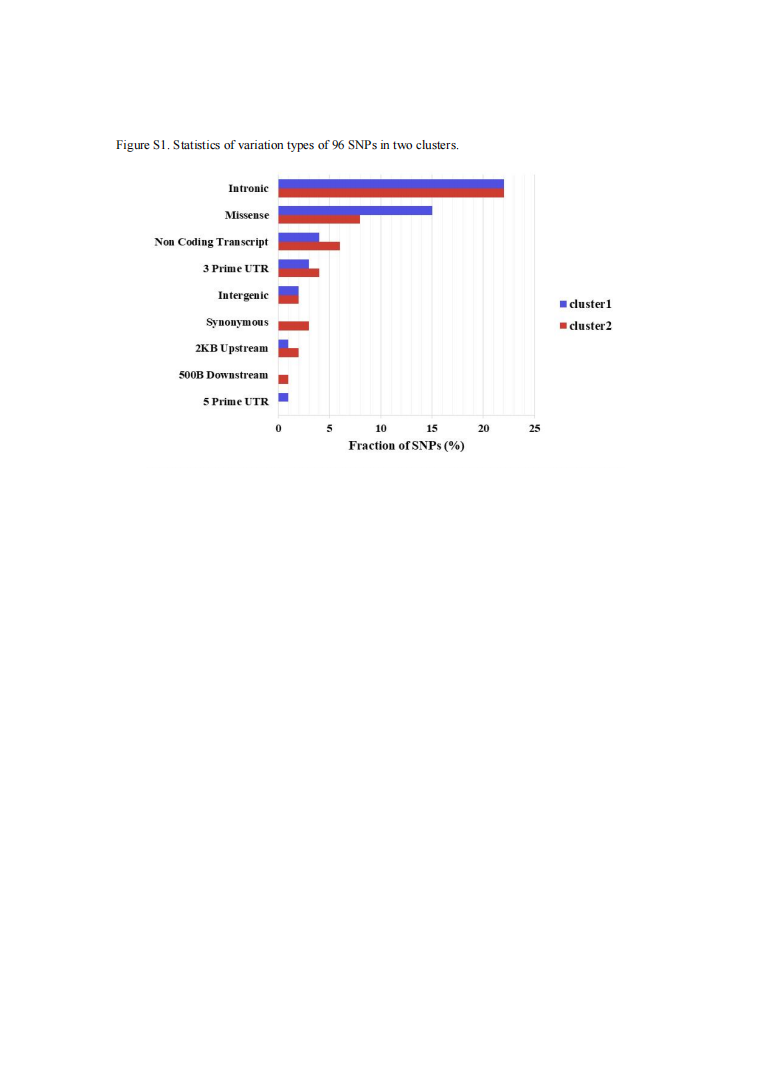

Supplement: Supplementary file 2 [file Image_1.tif]

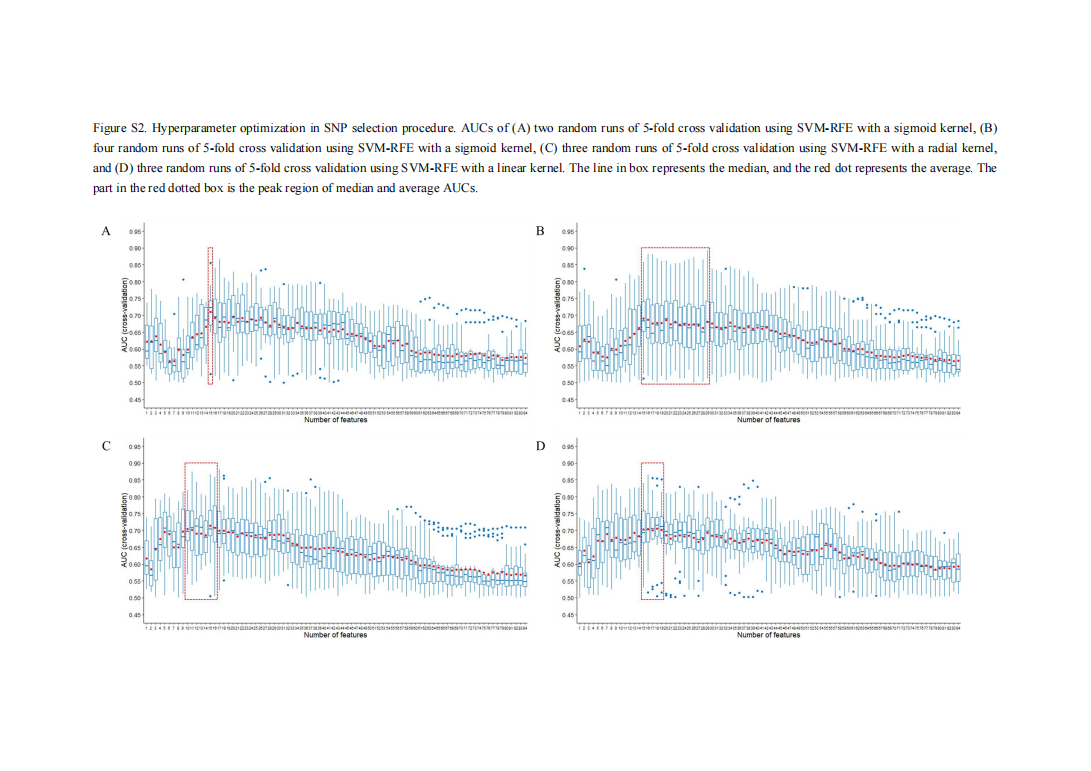

Supplement: Supplementary file 3 [file Image_2.tif]
